# Automatic Thinning Detection through Image Segmentation Using Equivalent Array-Type Lamp-Based Lock-in Thermography

**DOI:** 10.3390/s23031281

**Published:** 2023-01-22

**Authors:** Seungju Lee, Yoonjae Chung, Chunyoung Kim, Wontae Kim

**Affiliations:** 1 Department of Future Convergence Engineering, Kongju National University, 1223-24 Cheonan-Daero, Seobuk-gu, Cheonan-si 31080, Republic of Korea; 2 Eco-Sustainable Energy Research Institute, Kongju National University, 1223-24 Cheonan-Daero, Seobuk-gu, Cheonan-si 31080, Republic of Korea; 3 enesG, 8, Techno 10-ro, Yuseong-gu, Daejeon 34026, Republic of Korea

**Keywords:** array-type lamp, lock-in thermography, image segmentation, morphology operation, automatic detection, detectability evaluation

## Abstract

Among the non-destructive testing (NDT) techniques, infrared thermography (IRT) is an attractive and highly reliable technology that can measure the thermal response of a wide area in real-time. In this study, thinning defects in S275 specimens were detected using lock-in thermography (LIT). After acquiring phase and amplitude images using four-point signal processing, the optimal excitation frequency was calculated. After segmentation was performed on each defect area, binarization was performed using the Otsu algorithm. For automated detection, the boundary tracking algorithm was used. The number of pixels was calculated and the detectability using RMSE was evaluated. Clarification of defective objects using image segmentation detectability evaluation technique using RMSE was presented.

## 1. Introduction

Defects (discontinuity points) in metal structures are caused by various reasons, typically corrosion. For example, in the containment liner plant (CLP), there is a case in which voids are formed inside the concrete due to corrosion [1,2,3]. In addition, if corrosion occurs in structures such as bridges or plants, it may eventually lead to fracture and cause great damage. Therefore, efficient inspection techniques and condition monitoring techniques are required in real-time in order to minimize damage.

The process by which corrosion occurs is as follows. When structures are exposed to extreme environments, corrosion processes occur on their surfaces. When corrosion continues and time passes, local thinning occurs. As a result, it leads to fractures and causes significant damage. Corrosion present on the surface can be checked with the naked eye, so that it can be inspected simply by visual testing (VT) [4]. Areas that cannot be confirmed by VT are difficult, so it is possible to inspect defects that exist inside or on the back side by using non-destructive testing (NDT) techniques.

There are many types of NDT, and representatively, ultrasonic testing (UT) and radiography testing (RT) are used in many fields [5,6]. UT can precisely measure the inside thickness and enables fast local inspection, but the inspection area is small, so the overall inspection takes a long time [7,8]. RT has disadvantages that are harmful to the body [9]. In addition, eddy current testing (ECT), leak testing (LT), and microwave testing (MT) are being utilized.

In this study, the infrared thermography (IRT) technique was applied among NDT techniques that can efficiently inspect a large area in real-time [10,11,12]. IRT is a technique that uses an infrared camera to detect infrared energy naturally emitted by objects, convert it to temperature, and output a temperature distribution image in real-time [13,14,15]. It is possible to inspect a wide area in real-time and acquire or analyze high-level quantitative data [16].

There are many research cases on corrosion detection using the IRT technique. Doshvarpassand analyzed the entire literature on corrosion defect characteristics using an active IRT technique [17]. Kobayashi analyzed corrosion loss of reinforced concrete according to heating time using induction heating (IH) and IRT [18]. Cadelano compared and analyzed the SNR according to the heating and cooling stages using PCA and PPT data processing techniques [19]. R. Shrestha conducted a study to evaluate the size and depth of defects on the rear surface using the LIT technique [20].

A study on the detection of automatic thinning defects in the S275 specimen was conducted using the lock-in thermography (LIT) technique among the IRT method. Phase and amplitude images were acquired using the four-point signal process of LIT, and the optimal frequency was evaluated by calculating the SNR. Binarization of the segmented image was performed using the Otsu algorithm, and pixel noise was removed by performing the morphology operation. Automatic defect detection was performed using the boundary tracking algorithm, and detectability was evaluated using RMSE. In this study, an array-type halogen lamp device was developed to provide a uniform heat source. In addition, a clear object detection and detectability evaluation process technique through image segmentation was presented.

## 2. Theory

### 2.1. Theory of Four-Point Signal Process

The LIT is a technique in which a heat source in the form of a harmonic function is incident on an object, and a response signal generated at this time is processed to obtain changes in phase and amplitude [21,22,23]. When the heat source energy reaches the object’s surface, it is absorbed, and the phase shifts. When the energy reaches an area within an object whose thermophysical properties are non-uniform, the incident energy is partially reflected. The reflected energy interferes with the energy incident on the surface of the object, causing an interference pattern of the local surface temperature vibrating at the same frequency as the heat wave. For a planar plate, a 2D temperature field with a heat wave could be expressed as

(1) $$\frac{\partial T}{\partial t}=\frac{k}{{\rho c}_{p}}\frac{\partial^{2}T}{\partial x^{2}}$$

where T is the temperature, t is the time, k is the thermal conductivity coefficient, ρ is the density, $c_{p}$ is the specific heat, and x is the distance in the direction of heat flow. Equation (1) heated by the harmonic function can be expressed as follows.

(2) $$T\left(x,t\right)=T_{0}e^{-\frac{x}{\mu }}\cos\left(\omega t-\frac{x}{\mu }\right)$$

(3) $$\mu =\sqrt{\frac{2\alpha }{\omega }}=\sqrt{\frac{\alpha }{\pi f}}$$

(4) $$\alpha =\frac{k}{{\rho c}_{p}}$$

where $T_{0}$ is the initial temperature generated by the heat source, ω is the modulation excitation frequency, μ is the penetration depth, α is the thermal diffusion coefficient, and f is the frequency.

**Figure 1:**
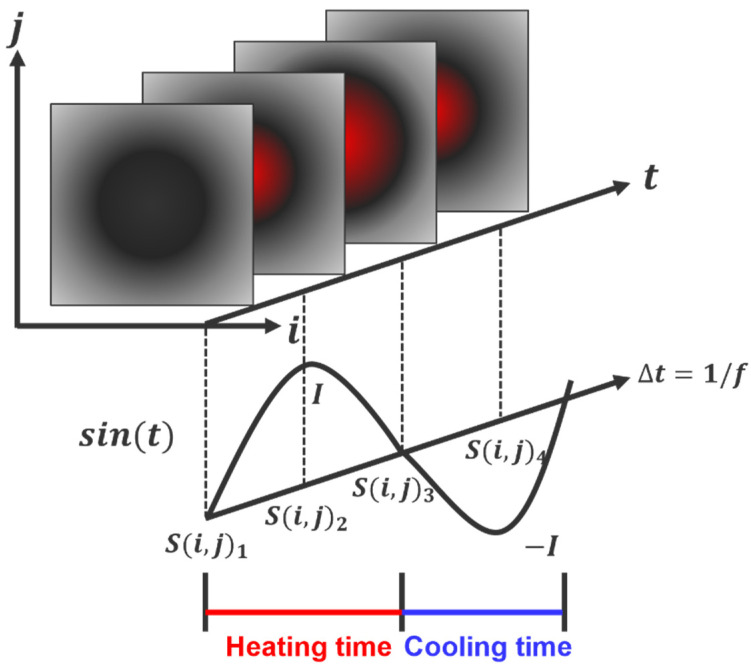
Principle of four-point signal processing to acquire phase and amplitude in LIT technique.

In the LIT technique, a heat source in the form of an external sine wave is incident on the plate surface, and the response temperature signal uses an infrared system to record 2D image data in real-time. The four-point method is a process used to convert phase and amplitude data. Figure 1 shows the principle of the four-point method processing. When there are four constant distance temperature data $S_{1}$, $S_{2}$, $S_{3}$, and $S_{4}$, the phase (∅) and amplitude (A) are as follows [24,25].

(5) $$\emptyset =\tan^{-1}\left(\frac{S_{1}-S_{3}}{S_{2}-S_{4}}\right)$$

(6) $$A=\sqrt{{\left(S_{1}-S_{3}\right)}^{2}+{\left(S_{2}-S_{4}\right)}^{2}}$$

The reflected heat wave is determined by the phase, amplitude, and modulated frequency. The principle of defect detection is based on the fact that the defective area has a phase delay with respect to the sound area. The phase delay is the result of the different thermal properties of the defective area and the sound area of the material. When the four-point signal processing is performed, the defect is detected more clearly. One of the main reasons the defective area is well defected is that the phase image is less susceptible to non-uniform heating, surface emissivity change, or environmental reflection than the raw thermal image. The excitation frequency of a heat wave has a direct correlation with the phase delay. In order to generate enough visible phase delay, an appropriate excitation frequency must be selected. In other words, using a low frequency with a long wavelength can detect deep defects, and using a high frequency with a short wavelength can detect shallow defects.

### 2.2. Binary Process Using Otsu Algorithm

**Figure 2:**
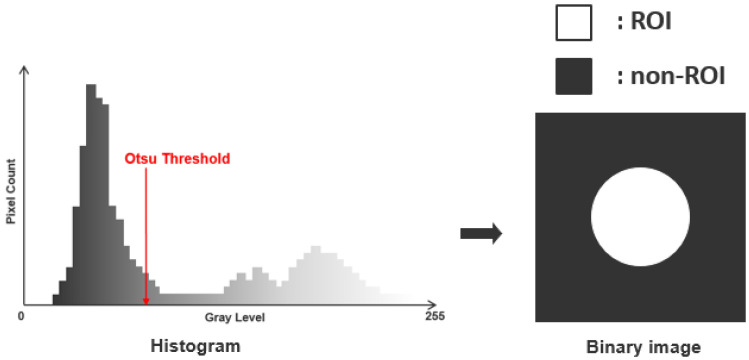
The principle of acquiring binary image by using gray scale-based histogram in the Otsu algorithm.

The Otsu algorithm is a technique that uses a histogram of a gray-level scale to calculate the optimal threshold for classifying contrast values as 0 and 1 [26,27,28]. In the gray-scale range (0−L), 0 to k are classified as intensity value 0, and k + 1 to L are classified as intensity value 1. Figure 2 shows the process of acquiring a binary image using the Otsu algorithm. Binarized images have the advantage of being able to clearly recognize defective objects.

In order to classify intensity values, the optimal threshold value must be calculated [29,30]. If it is an M × N image with L intensity levels such as 0, 1, 2, …, L−1, pixels with intensity values within [0, k] are classified as class 1, and intensity values within [k + 1, L + 1] are classified as class 2. The probability that a pixel is classified into class 1 or 2 is as follows.

(7) $$P_{1}\left(k\right)=\overset{k}{\underset{i=0}{\sum }}p_{i}$$

(8) $$P_{2}\left(k\right)=1-P_{1}\left(k\right)$$

The average intensity values of pixels classified into contrast values 0 and 1 are as follows.

(9) $$m_{1}\left(k\right)=\frac{1}{P_{1}\left(k\right)}\overset{k}{\underset{i=0}{\sum }}{\mathrm{iP}}_{i}$$

(10) $$m_{2}\left(k\right)=\frac{1}{P_{2}\left(k\right)}\overset{L-1}{\underset{i=k+1}{\sum }}{\mathrm{iP}}_{i}$$

There are mean intensity values up to the k level, which of all images is

(11) $$m_{G}=P_{1}m_{1}+P_{2}m_{2}$$

In order to calculate the optimal threshold value, the Otsu algorithm should find the maximum variance. The equation of between-class variance is as follows.

(12) $$\sigma_{b}^{2}=\frac{\{{\left(m_{G}P_{1}-m\left(k\right)\right\}}^{2}}{P_{1}\left(1-P_{1}\right)}$$

Although there are many types of algorithms or functions capable of performing binarization processing, the Otsu algorithm has the advantage of being able to perform binarization processing in a fast time by calculating a threshold value in real-time.

## 3. Experimental Setup

### 3.1. S275 Specimen

**Figure 3:**
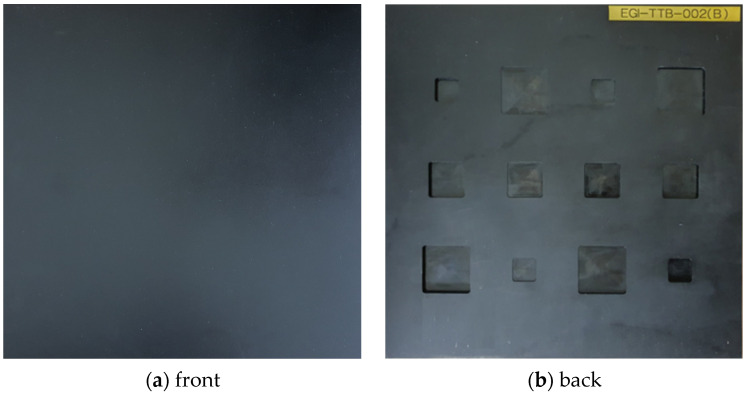
Front and back of S275 specimen with thinning defects.

**Figure 4:**
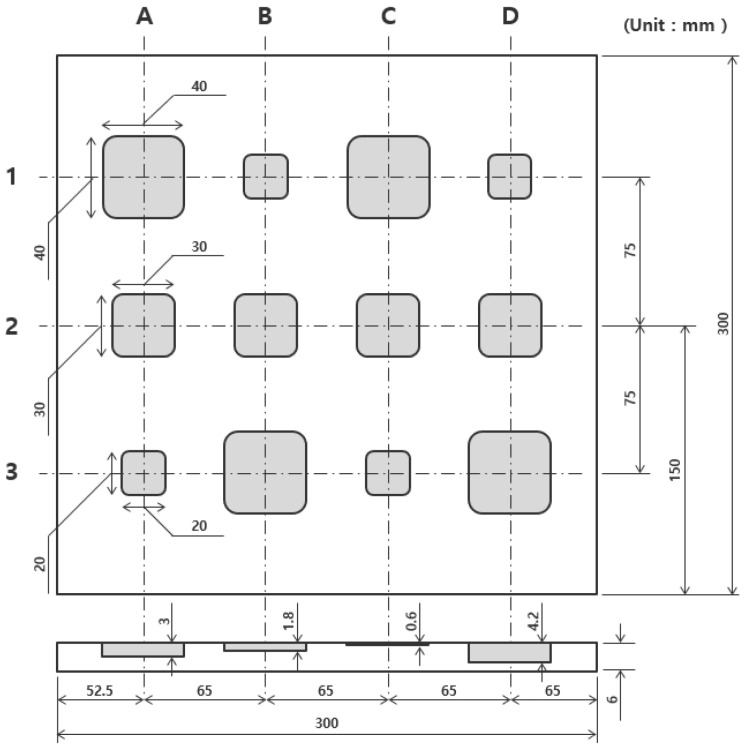
Dimensions of S275 specimen with various sizes of thinning defects.

The material of the specimen used in this study is S275, and Figure 3 shows the front and back of the specimen. The front side of the specimen was coated with KRYLON’s black paint to maintain an emissivity of 0.95 or more. Figure 4 shows the dimensions of the S275 specimen. There are a total of 12 artificial thinning defects, and the depth of the column axis is the same. The thinning depth consisted of 10%, 30%, 50%, and 70% of the total thickness. There are a total of 12 thinning defects, and it consists of a regular arrangement. The size of the specimen is 300 × 300 mm, and the thickness is 6 mm. The size of the defect is 40 × 40 mm, 30 × 30 mm, and 20 × 20 mm. Table 1 shows the properties of S275 material.

### 3.2. Experimental Setup of LIT System

**Figure 5:**
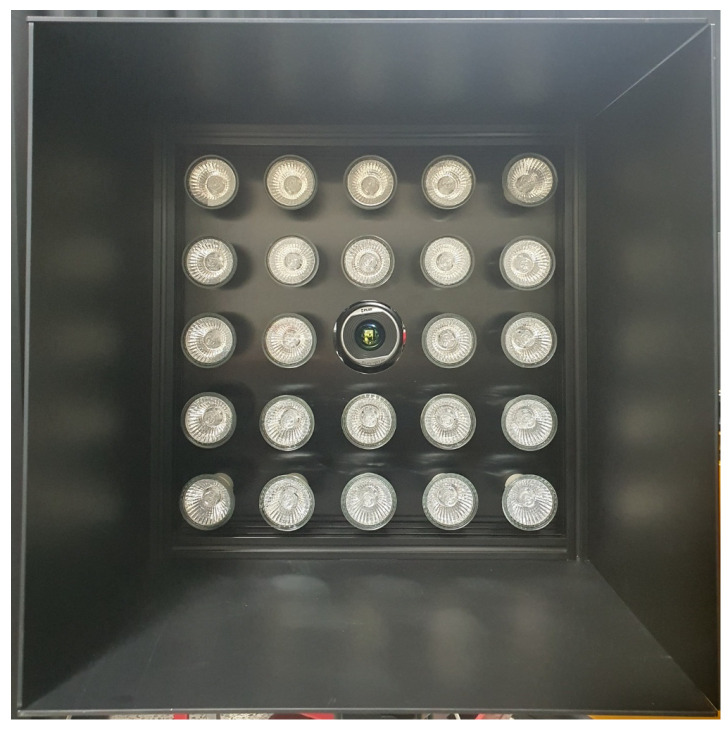
Halogen lamp device of 5 × 5 array type. Infrared camera placed in the center.

In this study, an array-type halogen lamp device was developed to supply a uniform heat source to the specimen. Figure 5 shows an array type halogen lamp. It is composed of lamps in a 5 × 5 arrangement, and a cover plate is additionally installed to minimize the influence of the surrounding environment. An infrared camera is located in the center of the array of lamps. The maximum output was 1.2 kW, and the distance between the specimen and the lamp was placed at 500 mm.

**Figure 6:**
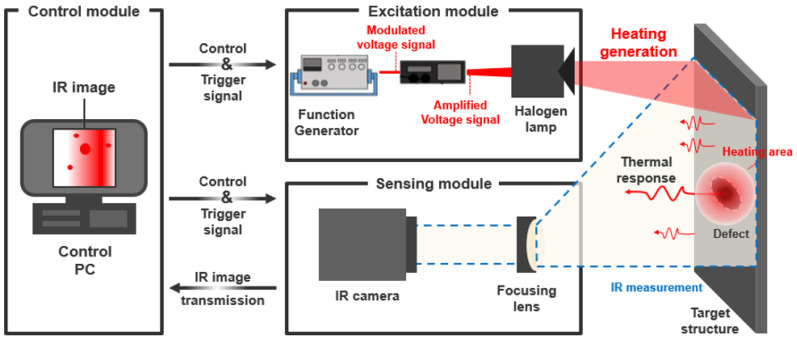
Schematic diagram of the LIT experimental setup for measuring the thermal response of specimen with thinning defects.

Figure 6 shows the LIT system of this study. The infrared camera is FLIR’s SC645 model (uncooled, 640 × 480 pixels, 7.5~13 μm, 50 Hz) and is used to measure the surface response of the specimen generated by the heat of the halogen lamp. The distance between the specimen and the infrared camera is 500 mm, which is the same as that of the lamp. The function generator (Agilent 33210A, Petaling Jaya, Malaysia) and the power amplifier were utilized to control the halogen lamp. The voltage range of the power amplifier is 0~10 V, and it was set to 10 V in this study. The range of the excitation frequency set in this study is 0.01~0.1 Hz and is increased by 0.01 Hz. The field of view (FOV) of the infrared camera is 25° (H) × 19° (V), and the focal length is 24.6 mm. The thermal response was measured in real-time using the dedicated software FLIR R&D of the infrared camera, and MATLAB software was used to analyze the thermal image.

## 4. Data Results of LIT

### 4.1. Images with Four-Point Signal Process

**Figure 7:**
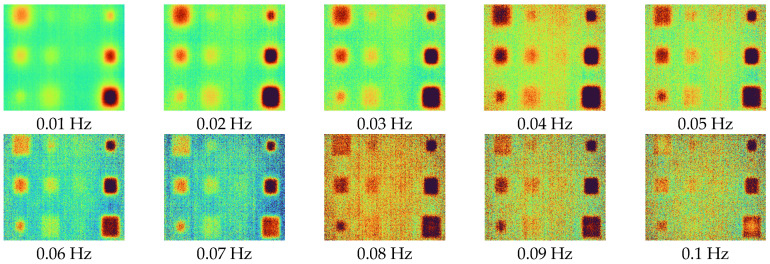
Phase images for excitation frequencies range from 0.01 to 0.1 Hz.

**Figure 8:**
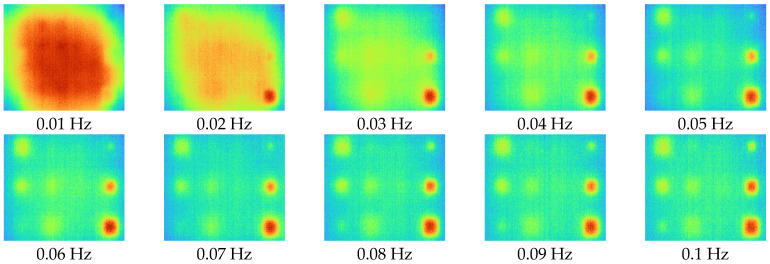
Amplitude images for excitation frequencies range from 0.01 to 0.1 Hz.

After acquiring 2D thermal images for each excitation frequency range using LIT infrared system equipment, phase and amplitude images were acquired using the four-point signal process. Figure 7 and Figure 8 show phase and amplitude images. In the case of a phase image, more noise can be identified as the frequency increases. In the case of the amplitude image, the lower the frequency, the larger the heat source provided, making it difficult to identify defects.

In order to calculate the optimal frequency in the set excitation frequency range, the SNR of the ROI (5 × 5 pixels) in the D3 defect was calculated, the equation is as follows [31].

(13) $$\mathrm{SNR}=20\log_{10}\left(\frac{\left|{\mathrm{DROI}}_{\mathrm{mean}}-{\mathrm{SROI}}_{\mathrm{mean}}\right|}{\sigma }\right)$$

where ${\mathrm{DROI}}_{\mathrm{mean}}$ and ${\mathrm{SROI}}_{\mathrm{mean}}$ are the arithmetic mean of all the pixels in the defective area and the sound area, respectively, and σ is the standard deviation of all the pixels in the sound area.

**Figure 9:**
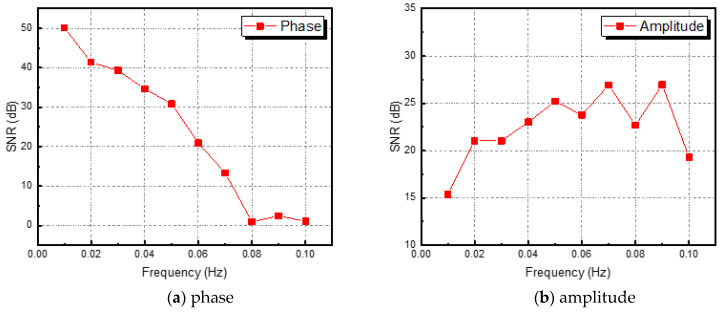
SNR values of phase and amplitude for excitation frequencies range from 0.01 to 0.1 Hz for optimal excitation frequency analysis.

Figure 9 shows the SNR values of phase and amplitude for a range of frequencies. The optimal excitation frequencies for phase and amplitude can be identified as 0.01 Hz and 0.09 Hz, respectively. Referring to Figure 7 and Figure 8, it can be seen that qualitatively, the lower the frequency of the phase and the higher the frequency of the amplitude, the more advantageous it is to identify defects. In addition, it can be confirmed that the phase and amplitude are inversely proportional.

### 4.2. Data Segmentation for Detection Improvement

**Figure 10:**
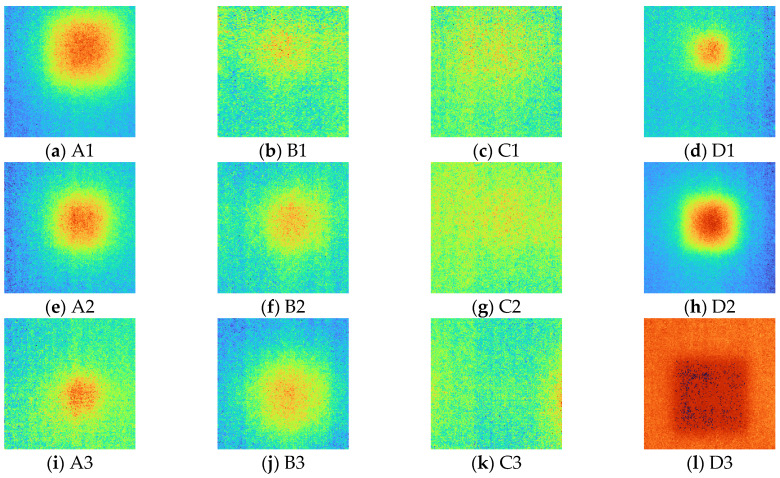
Segmentation image for phase data at 0.01 Hz.

**Figure 11:**
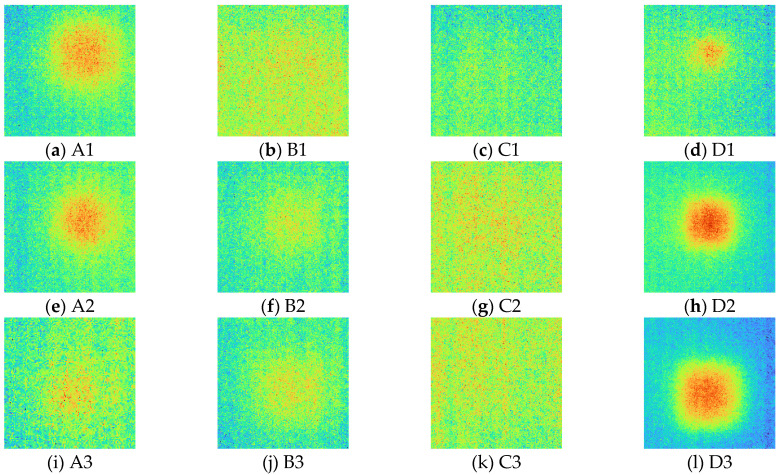
Segmentation image for amplitude data at 0.09 Hz.

In this study, segmentation was performed for all thinning defect before binarization using the Otsu algorithm. Figure 10 and Figure 11 show the segmentation images with phase of 0.01 Hz and amplitude of 0.09 Hz. A total of 12 image segmentations were performed for a total of 12 defect areas. The scale for the segmentation area was set to the same resolution of the infrared camera. In both the phase and amplitude images, defects in column C with a thinning depth of 10% are difficult to visually identify.

**Figure 12:**
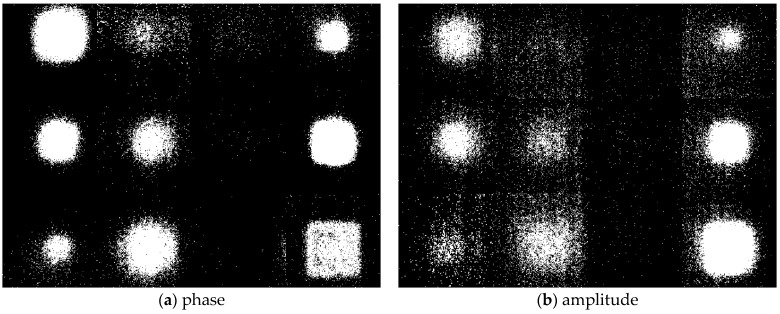
Binary images of phase and amplitude data using the Otsu algorithm.

The binarization of the segmentation image was performed using the Otsu algorithm, and then they were merged into a single image. Figure 12 shows the merged binary image of phase and amplitude. Binarization was performed after calculating all threshold values for each segmentation image. By converting the RGB scale to the gray-scale, visually clear defect objects can be identified. However, there is still a lot of noise, so it needs to be removed through a post-processing process. Further, like phase and amplitude images, it is difficult to identify defects in the C column, even in the binary image.

### 4.3. De-Noising Using Morphology Operation

**Figure 13:**
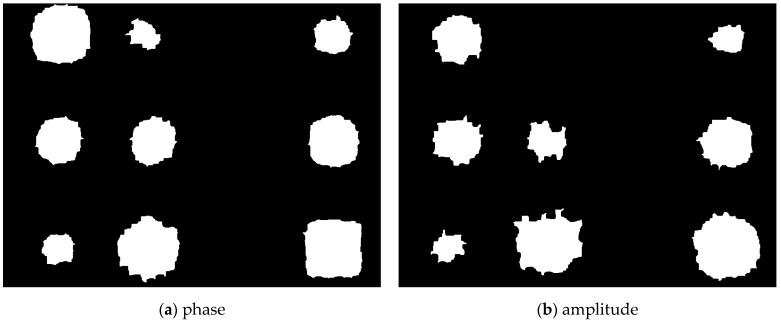
Binary images with de-noising performed using morphology operation.

The morphology operation was performed for de-noising existing in the binary image. Morphology calculation was performed through three steps as follows. First, de-noising was performed using the ‘bwareaopen’ function. Second, the ‘imclose’ function was used to fill the empty space with pixels. Third, the process of determining the contrast values of neighboring pixels was performed using the ‘bwmorph’ function. Figure 13 shows images of phase and amplitude with morphology operation applied. By applying the morphology operation, it is possible to confirm an image in which noise is effectively removed. Comparing the phase and amplitude images, the boundary of the object is relatively more uniform in the object of the phase image.

### 4.4. Automatic Detection of Thinning Defects

**Figure 14:**
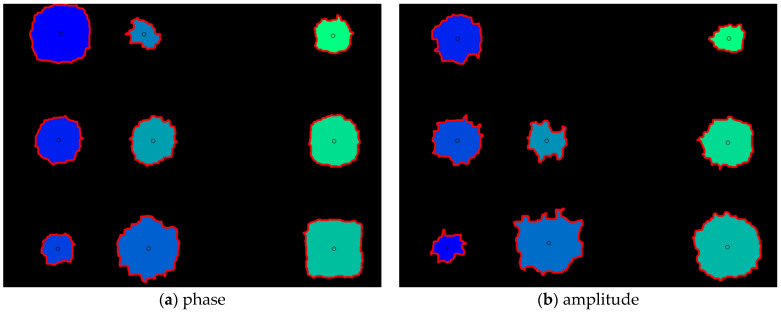
Images of phase and amplitude with automatic defect detection using boundary tracking algorithm.

The boundary tracking algorithm was utilized for the automatic detection of thinning defects. The boundary tracking algorithm is a technique for tracking the boundary of the object existing in the binary image. Figure 14 shows images of phase and amplitude with applied automatic detection. A total of 9 defects in phase and 8 defects in amplitude were detected. However, all defects in 3 columns failed to be detected. This can be considered as a reason for the low thermal contrast of the defective area and the sound area due to thinning of 10%. It is possible to qualitatively confirm that the boundary of the defect object in the phase image is relatively uniform compared to the amplitude.

### 4.5. Comparison Evaluation of Detectability

Detectability evaluation was performed using root mean square error (RMSE) on the binary image of phase and amplitude. The difference between the real value and the estimated value for each object was calculated, and the number of pixels with the contrast value of 1 in the binary image was calculated. The equation for RMSE is [17]

(14) $$\mathrm{RMSE}\left(\theta_{1},\theta_{2}\right)=\sqrt{\frac{\sum_{i=1}^{n}{\left(\theta_{1,i}-\theta_{2,i}\right)}^{2}}{n}}$$

where $\theta_{1}$ is the real value, $\theta_{2}$ is the estimated value, and n is the number of defect areas.

Table 2 shows the RMSE results of phase and amplitude. Defects not detected in the binary image were excluded. As confirmed qualitatively in Figure 14, it can be confirmed that the RMSE of the phase is calculated relatively low, and the detectability is high.

## 5. Conclusions and Future Works

In this study, the automatic detection of thinning defects in the S275 specimen was performed using the LIT technique based on array type halogen lamp. Image segmentation was performed for clear recognition of defective objects. Automatic defect detection using the algorithm and comparative analysis of detectability was performed. The main conclusions are as follows.

1. Phase and amplitude images were acquired using the four-point signal process of the LIT technique. The optimal excitation frequency was evaluated using SNR, and the phase was derived as 0.01 Hz and the amplitude as 0.09 Hz;
2. After image segmentation, the binary image was acquired using the Otsu algorithm. Pixel noise was removed by performing the three-step morphological calculation;
3. Automatic defect detection of phase and amplitude images was performed using the boundary tracking algorithm. A total of 9 defects in phase and 8 defects in amplitude were detected;
4. The detectability was evaluated by calculating the RMSE based on the number of pixels with the contrast value of 1 in the object area. Better results can be seen in phase than the amplitude.

Future work will be conducted on automatic defect detection of real-time images including full frames.

## Figures and Tables

**Table 1:** The material properties of S275 specimen.

Thermal Conductivity	50 W/m·K
Specific Heat	470 J/kg·K
Density	$7900\;\mathrm{kg}/m^{3}$
Initial Temperature	25 ℃

**Table 2:** RMSE values for phase and amplitude images.

Defect	Real Pixel Values	Estimated Pixel Values	
		Amplitude	Phase
A1	11,778	8081	13,111
A2	7078	7629	7583
A3	3636	2971	3470
B1	3636	-	2861
B2	7078	4771	7502
B3	11,778	14,131	12,995
C1	11,778	-	-
C2	7078	-	-
C3	3636	-	-
D1	3636	3217	4769
D2	7078	9165	9502
D3	11,778	15,239	13,788
RMSE		65.418	61.117

## Data Availability

Not applicable.
